# A Phylogenetic and Phenotypic Analysis of *Salmonella enterica* Serovar Weltevreden, an Emerging Agent of Diarrheal Disease in Tropical Regions

**DOI:** 10.1371/journal.pntd.0004446

**Published:** 2016-02-11

**Authors:** Carine Makendi, Andrew J. Page, Brendan W. Wren, Tu Le Thi Phuong, Simon Clare, Christine Hale, David Goulding, Elizabeth J. Klemm, Derek Pickard, Chinyere Okoro, Martin Hunt, Corinne N. Thompson, Nguyen Phu Huong Lan, Nhu Tran Do Hoang, Guy E. Thwaites, Simon Le Hello, Anne Brisabois, François-Xavier Weill, Stephen Baker, Gordon Dougan

**Affiliations:** 1 Wellcome Trust Sanger Institute, Wellcome Trust Genome Campus, Hinxton, Cambridgeshire, United Kingdom; 2 The London School of Hygiene and Tropical Medicine, London, United Kingdom; 3 The Hospital for Tropical Diseases, Wellcome Trust Major Overseas Programme, Oxford University Clinical Research Unit, Ho Chi Minh City, Vietnam; 4 The Department of Medicine, University of Cambridge, Cambridge, United Kingdom; 5 Centre for Tropical Medicine, Oxford University, Oxford, United Kingdom; 6 Institut Pasteur, Unité des Bactéries Pathogènes Entériques, Paris, France; 7 Université Paris-Est, ANSES, Laboratoire de Sécurité des Aliments, Maisons-Alfort, France; Massachusetts General Hospital, UNITED STATES

## Abstract

*Salmonella enterica* serovar Weltevreden (*S*. Weltevreden) is an emerging cause of diarrheal and invasive disease in humans residing in tropical regions. Despite the regional and international emergence of this *Salmonella* serovar, relatively little is known about its genetic diversity, genomics or virulence potential in model systems. Here we used whole genome sequencing and bioinformatics analyses to define the phylogenetic structure of a diverse global selection of *S*. Weltevreden. Phylogenetic analysis of more than 100 isolates demonstrated that the population of *S*. Weltevreden can be segregated into two main phylogenetic clusters, one associated predominantly with continental Southeast Asia and the other more internationally dispersed. Subcluster analysis suggested the local evolution of *S*. Weltevreden within specific geographical regions. Four of the isolates were sequenced using long read sequencing to produce high quality reference genomes. Phenotypic analysis in Hep-2 cells and in a murine infection model indicated that *S*. Weltevreden were significantly attenuated in these models compared to the classical *S*. Typhimurium reference strain SL1344. Our work outlines novel insights into this important emerging pathogen and provides a baseline understanding for future research studies.

## Author Summary

Organisms belonging to the species *Salmonella enterica* are a major cause of infection globally. Such infections can be zoonotic in origin or transmitted between humans. One of the most notable features of the genus *Salmonella* is that the dominant serovars that cause human infections change over time, with new threats periodically emerging. These trends often go unnoticed and are underreported in low-income locations. There is good evidence that *Salmonella* Weltevreden is emerging (particularly in low-income countries in the tropics) as a significant cause of diarrhea and sometimes invasive bacterial disease in humans. However, little is known about the phylogenetic structure or virulence potential of this unstudied serovar. Here, we provide a detailed phylogenetic analysis of *S*. Weltevreden through whole genome sequencing and bioinformatics tools. We identify that specific phylogenetic clusters are associated with geographical regions, providing novel data regarding the global distribution of different clades and high quality reference genomes to facilitate future work. We additionally show that *S*. Weltevreden has a distinct virulence-associated phenotype in conventional laboratory *Salmonella* pathogenicity assays that will guide future investigations on this serovar.

## Introduction

*Salmonella enterica* is a globally distributed Gram-negative enteric bacterial species that is responsible for significant levels of morbidity and mortality in both humans and animals [1]. *S*. *enterica* is currently classified into six subspecies and >2,500 serovars on the basis of the White- Kauffmann-Le Minor scheme that exploits specific typing sera against O (lipopolysaccharide) and H (flagella) antigens [2]. *S*. *enterica* organisms can also be loosely assigned into the so called typhoidal or non-typhoidal *Salmonella* (NTS) serovars. Typhoidal *Salmonella* are typically adapted to cause systemic disease in humans e.g. *Salmonella enterica* serovar Typhi (*S*. Typhi) the cause of the typhoid fever [3,4]. In contrast, NTS are more frequently, but not exclusively, associated with localized gastroenteritis and are more promiscuous and zoonotic than typhoidal serovars, and can infect multiple hosts e.g. *S*. Typhimurium [5,6]. Genetically, *S*. *enterica* is regarded as a broad and ancient species; different serovars/isolates can vary by 100,000s of single nucleotide polymorphisms (SNPs) and contain a variable array of genomic islands and prophages [7,8]. Further, many *Salmonella* serovars are monomorphic or clonal [6,9], however, there can be significant genetic diversity within a specific serovar e.g. *S*. Typhimurium [10] and generally studies on genetic diversity within a particular serovar are scarce, particularly using newer high-throughput approaches such as whole genome sequencing.

Most human NTS infections are commonly regarded as having a zoonotic source, at least in developed countries [11]. Some serovars, such as *S*. Cholerasuis, are predominantly associated with a particular mammalian host (pigs in the case of *S*. Cholerasuis) whereas others are more promiscuous. Indeed, the sources of many outbreaks of human salmonellosis are difficult to trace and often remain unidentified. In cases where the source is known, these have commonly been associated with raw or undercooked meat, eggs, vegetables or contamination has occurring during processing [12,13]. Notably, the frequency of outbreaks associated with particular serovars can vary in terms of source, incidence and geographical distribution. *Salmonella enterica* serovar Weltevreden (*S*. Weltevreden) is being increasingly reported as a cause of diarrheal disease in humans [14,15]. This little described serovar is emerging as a significant food-borne pathogen in Asia where it is reported to be associated with fish or aquatic food production systems [14]. *S*. Weltevreden has also been reported in Europe; an outbreak of diarrhea in Scandinavia was linked to alfalfa sprouts contaminated with *S*. Weltevreden [16]. Whole genome sequencing (WGS) has been used to define structure within various populations of bacterial pathogens, including some of the key serovars of *S*. *enterica* [17,18]. Genome sequences of four *S*. Weltevreden isolates have been previously decoded [19,20], but information regarding their phylogenic structure is limited. Here, we report a phylogenetic analysis of a global collection of *S*. Weltevreden; additionally providing complete reference genomes to facilitate future analysis. Furthermore, aiming to assess their pathogenic potential in comparison to other *Salmonella* we have phenotyped selected *S*. Weltevreden isolates in order to establish the value of particular experimental models.

## Methods

### DNA sequencing and assemblies

The 115 strains contributing to this study, their origins and their resulting accession numbers are described in S1 Table. All DNA samples were extracted using the wizard genomic DNA extraction kit (Promega, USA) and sequenced using the Illumina HiSeq 2500 platform. A reference genome of *S*. Weltevreden 10259 was generated using both Illumina and PacBio sequencing technologies. The assembly was built initially using the PacBio sequence reads then manually assembled. Raw sequencing reads of each sample were run through a Kraken database [21] (0.10.6) for taxonomic identification. All of the Illumina sequences were aligned to the complete reference genome generated from *S*. Weltevreden 10259. The reads, in FASTQ format, were first split into groups containing 1,000,000 reads. Each group of reads was individually aligned using SMALT (https://www.sanger.ac.uk/resources/software/smalt/) (0.7.4).

Aligned reads were merged using SAMtools [22] (version 0.1.19), coordinate sorted, and outputted as a BAM file. Optical duplicates were identified using Picard (http://broadinstitute.github.io/picard/) (1.9.2). Statistics regarding each mapping were generated using BamCheck [22] including read coverage of the reference genome, reads aligned, perfect pairs, unmapped reads and actual insert size. The resulting data was evaluated manually to identify poor quality sequencing data.

Illumina-generated sequences were assembled using a pipeline (https://github.com/sanger-pathogens/vr-codebase) developed at the Wellcome Trust Sanger Institute. For each genome, the *de novo* short-read assembler Velvet [23] (1.2.09) was used to generate multiple assemblies by varying the k-mer size between 66% and 90% of the read length using Velvet Optimiser (https://github.com/tseemann/VelvetOptimiser). From these, the assembly with the highest N50 was chosen. Contigs were excluded from the assembly if they were shorter than the target fragment size (400 bases). A scaffold assembly of the contigs was built by iteratively running SSPACE [24] (version 2.0) beginning with the contigs which were predicted to map next to each other. The reads were then mapped again to the scaffold assembly and perfect pairs were excluded. Next, gaps identified as one or more N’s, were targeted for closure by running 120 iterations of GapFiller [25] (version 1.11), using a decreasing read evidence threshold. Finally, the reads were aligned back to the improved assembly using SMALT (https://www.sanger.ac.uk/resources/software/smalt/) and a set of statistics was produced for assessing the quality of the assembly. PacBio raw read data for each sample was manually assembled using the PacBio SMRT analysis pipeline (https://github.com/PacificBiosciences/SMRT-Analysis/) (2.2). The raw unfinished assemblies all produced a single non-circular chromosome plus some other small contigs, some of which were plasmids or unresolved assembly variants. If the ends of a contig overlapped, they were identified as candidates for circularization using a protocol recommended by PacBio (https://github.com/PacificBiosciences/Bioinformatics-Training/wiki/Circularizing-and-trimming). A virtual break was manually introduced into the chromosome sequence at the *thrA* gene, to match the starting point of other published *S*. *enterica* references. Plasmids were also artificially broken at the replication gene. The sequences were then circularized using the genome assembler, Minimus [26] (version 2 part of AMOS version 3.1), which removed the overlapping sequence. Quiver was then used by the circularized sequence and the raw reads to correct errors in the circularized region. As high quality short read data from Illumina were available, ICORN2 (version 0.97) was used to correct minor errors in the assembly, providing a very high quality reference sequence, as assessed by REAPR [27] assembly was subsequently annotated with Prokka [28]. All sequences and assemblies are freely available; accession numbers are provided in S1 Table.

### Phylogenetic analysis

SNPs were called on each set of aligned reads using mpileup with the parameters ‘samtools mpileup -d 1000 -DSugBf ref bam’. The raw SNPs were then passed into BCFtools and were filtered into a higher quality set. A virtual pseudo-genome was then constructed by substituting the base call at each site (variant and non-variant) into the reference genome. For a SNP to be called the depth had to be greater than 4 reads, and be present on both strands, with at least 75% of reads containing the SNP at that position. The mapping quality had to be greater than 30 (less than 1 in 1000 probability that the mapping was incorrect). If a SNP failed to meet these criteria it is substituted with an ‘N’. Insertions with respect to the reference genome were ignored. Deletions with respect to the reference genome were filled up with ‘N’ characters in the pseudo-genome in order to keep it aligned and at the same length relative to the reference genome. Heterozygous sites were turned into homozygous alleles by selecting the first allele in the BCF file. However, if the first allele was an insertion or deletion (indel), the second allele in the BCF file was taken. If the second allele was also an indel, a single ‘N’ character was used. All of the pseudo-genomes were then merged into a single multi-FASTA alignment file, including the reference sequence.

Since the median fragment size for the isolates sequenced on Illumina was 400 bases, the reference genome was blasted against itself (2.2.31) in order to identify repeats larger than 400 bases. All bases falling within these regions were replaced with ‘N’ in the multi-FASTA alignment file and therefore not included in the analysis. The filtered multi-FASTA alignment was then checked for recombination using Gubbins [29] (1.3.4). Five iterations of Gubbins were run and in each iteration a phylogenetic tree was constructed with RAxML [30] with the GAMMA GTR model, and internal ancestral sequences were inferred using FastML [31] (version 3.1). Recombinant sequences were detected and a multi-FASTA alignment with the recombinant regions was masked out. This data was then used as the input to the next iteration. RAxML with 100 bootstraps was then run over the final multi-FASTA alignment to provide a high quality phylogenetic tree in newick format.

The population structure of the phylogenetic tree was validated using a Bayesian statistical approach. Hierarchical BAPS [32] (version 6.0) was used to perform a hierarchical clustering of the multi-FASTA alignment to reveal a nested genetic population structure. Once the clusters were identified, SNPs, which uniquely defined each of the clusters (in 100% of isolates in a cluster) were extracted using BioPericles (https://github.com/sanger-pathogens/BioPericles) (version 0.1.0). Exploiting the multi-FASTA alignment with recombination removed, a consensus sequence was generated for each cluster and any bases which varied or contained missing data were replaced by ‘N’. The consensus sequences were merged into a single multi-FASTA alignment file and SNP locations were identified using snp sites (https://github.com/sanger-pathogens/snp_sites) (version 2.0.1). Each SNP was then annotated using the reference annotation (10259) GFF3 file. An annotated VCF file was produced with VEP syntax [33] listing the type of change (intergenic/ synonymous/ nonsynonymous), the amino acid (before and after) and the amino acid position in the gene, along with the coordinates of each SNP relative to the reference genome, the reference base, the allele base and the presence and absence of the variant in each cluster. These cluster defining SNPs were then further annotated with the functional annotation of the gene they occurred in.

Antimicrobial resistance was predicted from each sample’s raw sequencing reads using ARIBA [34] (version 0.4.1), which performs antibiotic resistance identification by assembly and alignment. A manually curated input database of known resistance genes in FASTA format was used as input along with the paired end sequencing reads in FASTQ format. The resistance gene sequences were first clustered using CD-hit [35] (4.6). The raw reads were then aligned to a representative sequence for each resistance cluster. Reads which mapped and their complimentary strand equivalents were extracted. A local assembly was performed on the reads for each cluster (version 3.5), where the resistance genes for the cluster were used as ‘untrusted contigs’. This generates a candidate gene along with sequence on either side if the gene is present in the reads. MUMmer [36] (3.23) was then used to identify differences between the assembled contig and the known resistance gene and the results were reported along with any variation found and quality flags. These were manually inspected and samples with 100% matches to resistance genes and with a complete open reading frame were flagged as being potentially candidates for visual inspection.

A pan genome was constructed using Roary [37] (version 3.2.5) from the annotated assemblies of the sample set with a percentage protein identity of 95%. The protein sequences were first extracted and iteratively pre-clustered with cd-hit (version 4.6) down to 98% identity. An all against all blast (version 2.2.31) was performed on the remaining non-clustered sequences and a single representative sequence from each cd-hit cluster was selected. The data were used by MCL [38] (version 11–294) to cluster the sequences. The preclusters and the MCL clusters were merged and paralogs were split by inspecting the conserved gene neighborhood around each sequence (5 genes on either side). Each sequence for each cluster was independently aligned using PRANK [39] (version 0.140603) and combined to form a multi-FASTA alignment of the core genes.

### Hep-2 cells invasion assay

To facilitate the analysis of invasion assays, *S*. Typhimurium SL1344 and *S*. Weltevreden C2346, 10259, 98_11262 and 99_3134 were transformed with the plasmid pSsaG that directs the expression of GFP from the *ssaG* promoter [40]. Hep-2 cells were cultured in Glasgow’s minimal essential medium (GMEM, Sigma) supplemented with 2 mM L-Glutamate and 10% (volume/volume) heat- inactivated fetal bovine serum (FBS). Cells were seeded into 24-well plates (10^5^ cells per well) and cultured overnight. *Salmonella* were initially cultured at 37°C with agitation (250 rpm) in 5ml LB broth for 4.5 hours. An aliquot was then diluted 1:50 in LB broth and grown at 37°C overnight as a static culture to optimize *Salmonella* pathogenicity island 1 (SPI1) gene expression. For infection, the bacterial cultures were re-suspended in fresh GMEM media supplemented with 2 mM L-Glutamate and 10% (volume/volume) heat-inactivated FBS, in order to obtain a multiplicity of infection (MOI) 50. The MOI was confirmed by plating 10μl spots of 10-fold serial dilutions of the bacterial solution onto agar plates. After 30 minutes of incubation (to allow *Salmonella* invasion), cells were washed with phosphate-buffered saline before adding GMEM supplemented with gentamicin (50μg/ml). Cells were incubated for the appropriate length of time and then washed and lysed with 0.1% Triton X-100. Dilutions of the cell lysates were plated onto agar plates to determine the number of intracellular bacteria. Alternatively, cells were washed and fixed onto 13mm coverslips with 4% formaldehyde then stored in PBS for confocal or electron microscopy.

### Confocal microscopy

*Salmonella* infected cells were washed twice with the wash buffer from the Cytotoxicity 3 kit after fixation and permeabilized with the permeability buffer from the same kit for 10 minutes. The cells were then blocked with the block buffer for 20 min at room temperature and stained with goat anti-*Salmonella* CSA-1 antibody followed by tagged secondary antibody. Glass coverslips were mounted onto a microscopic slide along with ProLong Gold antifade reagent DAPI (Invitrogen). The preparations were observed with an LSM510 META confocal microscope (Zeiss).

### Murine infections

Six groups of five C57BL/6 mice were challenged intravenously with 2 x 10^3^ CFU of *S*. Typhimurium SL1344, *S*. Weltevreden C2346, 10259, 98_11262, 99_3134 or PBS as a control. The mice were followed for four days. They were all subsequently culled at day 4, or earlier if they were critically moribund. For the streptomycin infection model, six groups of five C57BL/6 mice each were pre-treated with 10 mg of streptomycin (200μl of a stock solution of 50mg/ml of streptomycin) 24 hours before challenge. The first group (naïve) was orally inoculated with PBS; the second, the third and the fourth groups were orally challenged with approximately 5.5x 10^5^ CFU of respectively *S*. Typhimurium SL1344, *S*. Weltevreden C2346, *S*. Weltevreden 10259, *S*. Weltevreden 98_11262 and *S*. Weltevreden 99_3134. The mice were sacrificed four days post challenge and caecum was removed from all mice for further analysis. Part of the caecum was used for histology to look for inflammation and the remaining part was plated on LB agar in order to check for bacterial colonization of the colon.

## Results

### A reference *Salmonella* Weltevreden genome in the context of Salmonella enterica

One hundred and fifteen *S*. Weltevreden isolates were collected from 18 countries encompassing South Asia, Southeast Asia and Oceania (S1 Table). These isolates were collected from a diverse range of sources including the environment, food, animal waste, human feces and blood. The collection of organisms spanned over 50 years (isolated between 1940 and 2013). DNA from all 115 *S*. Weltevreden isolates were sequenced using an Illumina platform and these sequences, together with four other previously published *S*. Weltevreden sequences [19,20], were assessed using the reference Multi-Locus Sequence Type database (http://mlst.warwick.ac.uk/mlst/dbs/Senterica) and confirmed to be Sequence Type (ST) 365. Table 1 summarizes the souce and the location of the isolates in this study.

**Table 1:** Source and location of *Salmonella* Weltevreden isolates contributing to this study.

Isolate characteristics	Number of isolates
**Source**	
Human stool	77
Human bloodstream	2
Food (meat/vegetables)	12
Food (seafood/fish)	9
Animals	10
Environment	3
Industrial (pig feed)	2
**Location**	
North Africa	1
Latin America	7
Europe	11
Oceania	17
Indian Ocean	22
South and Southeast Asia	56

**Fig 1:**
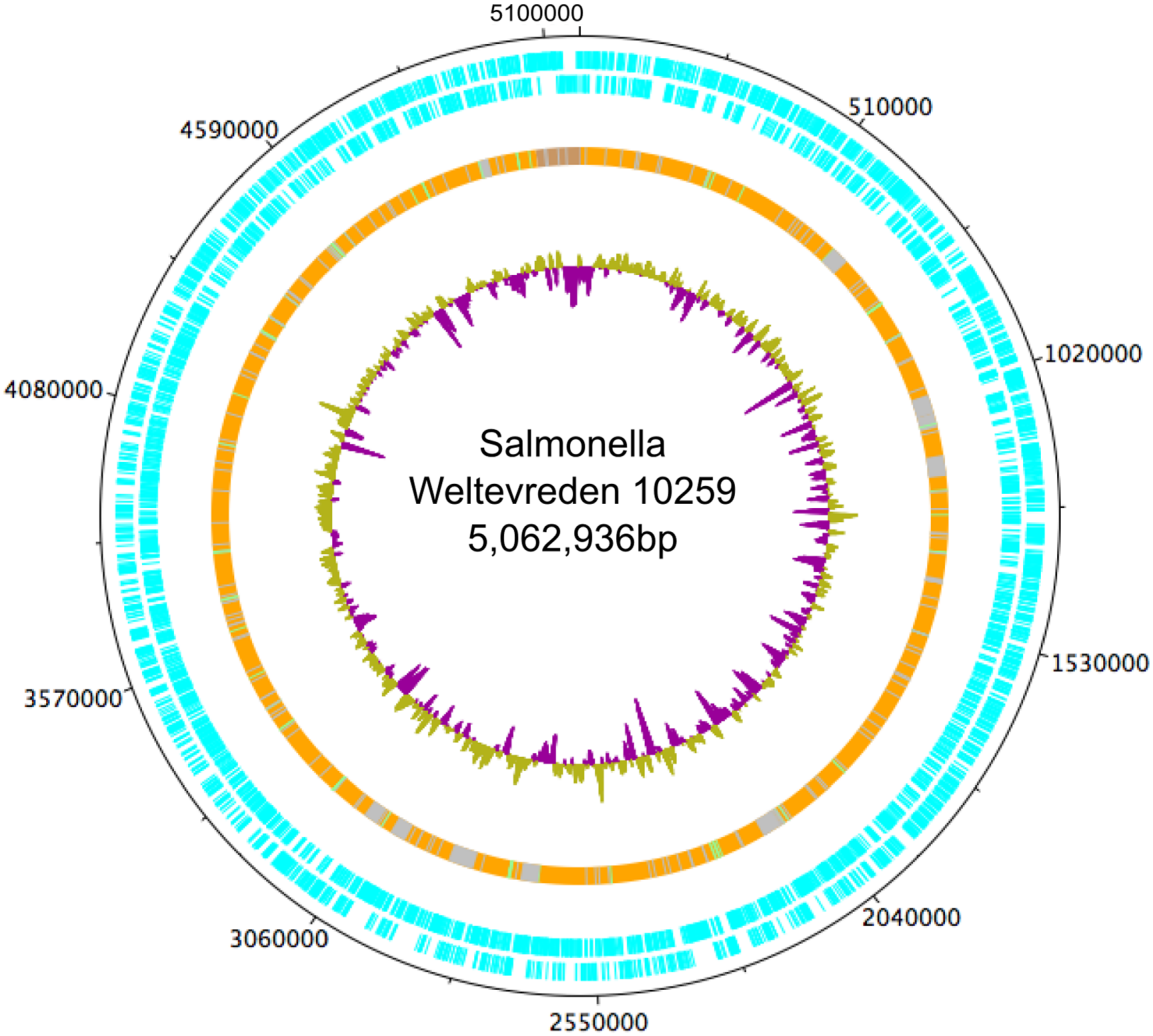
The chromosomal organization of Salmonella Weltevreden 10259. Circularized DNA plotter diagram of the chromosome of S. Weltevreden 10259, orientated from the origin; the outer black circle designating the genome base positions around the chromosome. The outer blue circles depict predicted 4,723 CDSs on both the forward and reverse strands. The predominantly orange circle represents the main chromosomal core structure with likely horizontally acquired DNA elements, including grey areas representing non-coding RNA (ncRNA) and green areas representing tRNA. The inner circle is a GC skew plot ((GC)/(G+C)), demonstrating evidence of historic lateral gene transfer.

In order to generate a complete reference genome for *S*. Weltevreden, DNA from the isolate 10259 obtained from a stool of a Vietnamese child with diarrheal disease [41], was sequenced using both the Illumina HiSeq and PacBio RSII long read sequence platforms. After manual adjustment, a single contig representative of the main *S*. Weltevreden chromosome was assembled, along with an additional contig representing a large plasmid. The chromosome of *S*. Weltevreden 10259 (Accession number LN890518) was a single circular molecule of 5,062,936 bps harboring 4,723 predicted coding DNA sequences (CDSs) with an average G+C content of 52.1% (Fig 1). The single plasmid, named pCM101 (Accession number LN890519), was 98,756 bps with 98 predicted CDSs. High quality (PacBio) reference genomes were generated for three additional isolates: C2346 (Accession numbers LN890520 and LN890521), 98_11232 (Accession numbers LN890522 and LN890523) and 99_3134 (Accession numbers LN890524 to LN890526).

**Fig 2:**
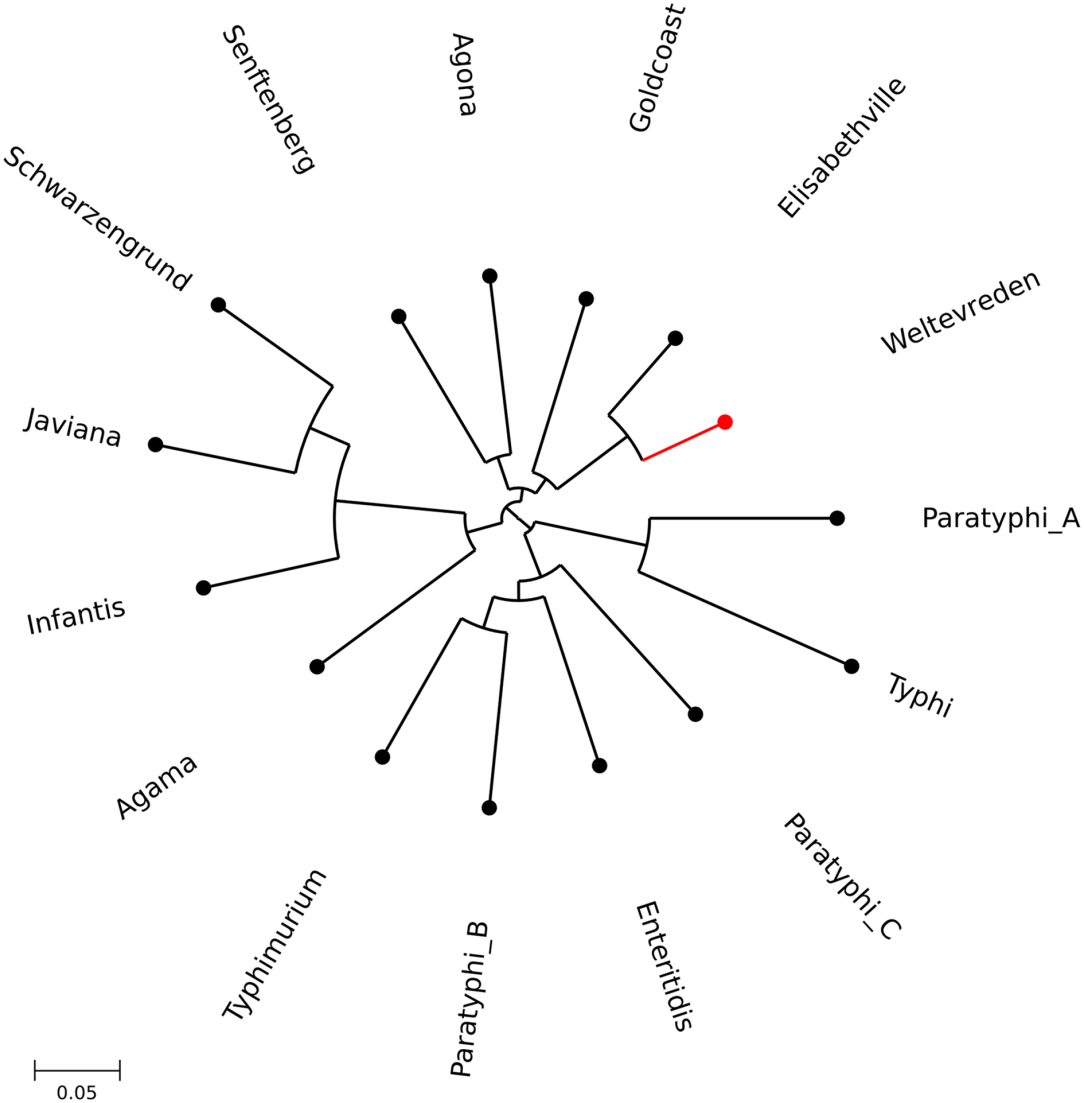
The phylogenetic positioning of Salmonella Weltevreden within Salmonella enterica. Maximum likelihood phylogenetic tree showing S. Weltevreden in the context of 14 additional serovars of Salmonella enterica. 3,269 core genes from whole genome sequences, representing 3,161,517 bps, were used to construct this tree; SNPs were detected at 134,485 positions. S. Weltevreden is highlighted in red and the scale is shown.

The contiguous *S*. Weltevreden 10259 reference genome was compared with 14 other isolates representative of the breadth across different *S*. *enterica* serovars. A single high quality assembly was chosen for each serovar from a publically available dataset and a pan genome was constructed [42]. A core *Salmonella* genome of 3,269 genes was identified, representing 3,161,517 bps, with SNPs at 134,485 positions. These data were used to create a phylogenetic tree to in which to position *S*. Weltevreden within the context of the broad species, *S*. *enterica* [43](Fig 2). The nearest phylogenetic neighbor to *S*. Weltevreden was *S*. Elisabethville with a difference of 16,693 bps in the core genes of the representative isolates. Notably, this observation was also supported by the similarities in their serological composition (*S*. Weltevreden; O:3, O:10 or 15 and r, z6 positive, *S*. Elisabethville; O:3, O:10 and r, 1,7 positive). *S*. Agona also mapped close to *S*. Weltevreden; this finding was in agreement with previously published data [20,43].

### The phylogenetic structure of *Salmonella* Weltevreden

**Fig 3:**
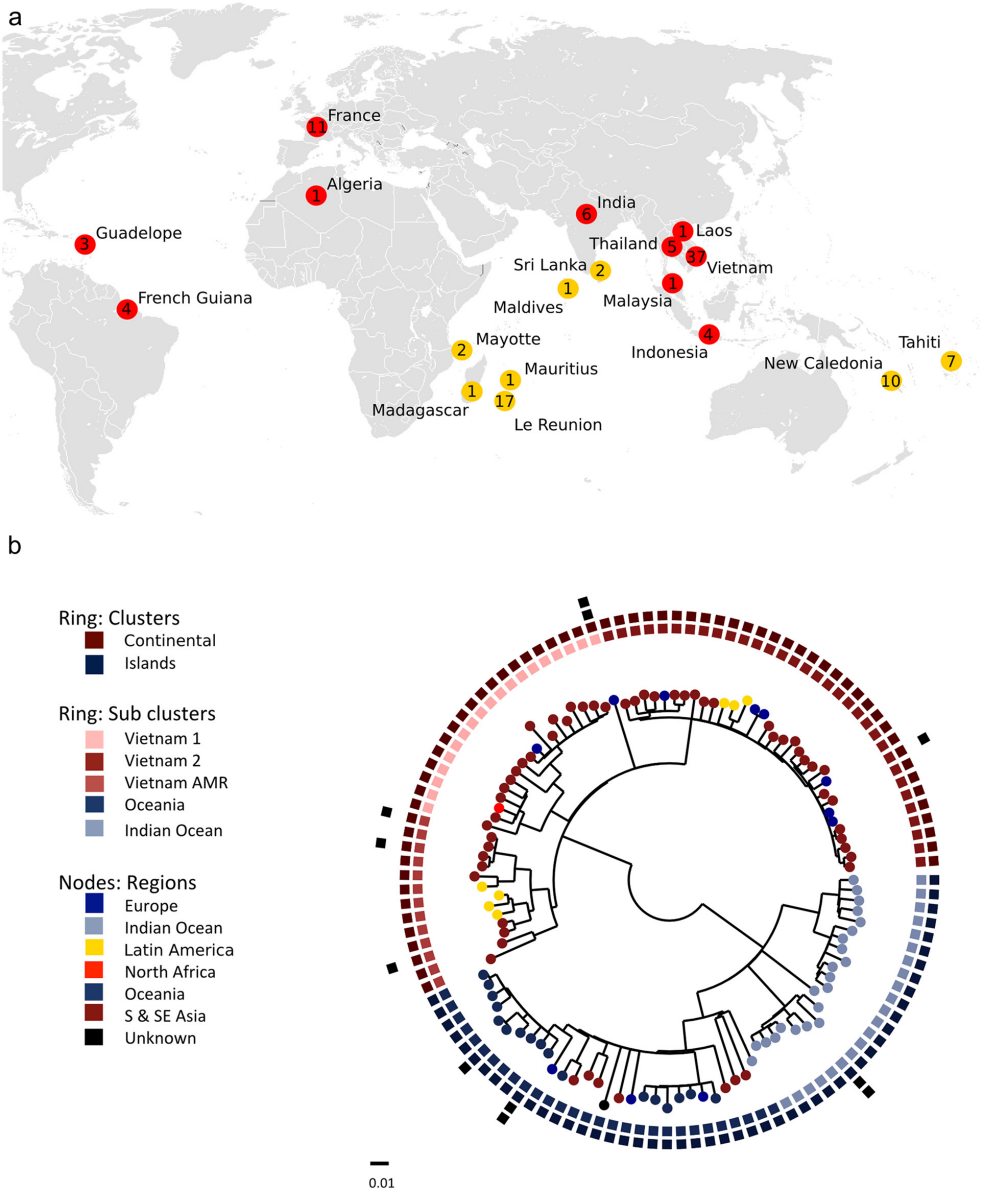
The population structure of Salmonella Weltevreden. a) The locations of sampling in which the 115 S. Weltevreden isolates included in the study were isolated. Each colored marker (red for ‘Continental cluster’ and yellow for ‘Island cluster’) represents the phylogenetic cluster present in the location and the number of organisms isolated in each sampled location. b) Maximum likelihood phylogenetic tree constructed using 2,601 SNPs showing the population structure of S. Weltevreden isolates with key metadata. The outer most ring shows isolates with predicted resistance against antimicrobials. The second ring shows isolates with laboratory confirmed resistance against antimicrobials. The third ring shows clusters (defined by BAPS) and the fourth ring shows the sub-cluster. Nodes are colored by geographical region.

Nothing is currently known about the phylogenetic organization with the single serovar of *S*. Weltevreden. Consequently, a phylogenetic tree was constructed using data from 115 sequenced *S*. Weltevreden. SNPs were present at 22,569 positions; these data were then filtered using Gubbins to refine the final phylogeny by removing fragments of the genome with a recombinant signal. A total of 218 recombination blocks were identified in the sample set (S1 Fig), which reduced the number of core SNPs to 2,601. The subsequent phylogenetic analysis identified two primary clusters, designated here as the ‘*Island Cluster’* and the ‘*Continental Cluster*’ correlating broadly with where the organisms were predominantly isolated (Fig 3). In total 112 SNPs discriminated between the two main clusters (S2 Table); these SNPs were found to distributed uniformly across the genome with no obvious high-density clusters. None of the identified mutations were found to be associated with premature stop codons. Indeed, our data suggest that in general pseudogene formation is not a dominant feature of the *S*. Weltevreden genome.

We were able to broadly subdivide *S*. Weltevreden into five subclusters, again correlating largely to where the organisms were isolated, or their hypothetical origin. The *Island Cluster* contains two subclusters, one drawn primarily from islands in the Indian Ocean (*Indian Ocean subcluster*) and the other from islands in Oceania or from nearby Southeast Asian countries (*Oceania subcluster*). The phylogenetic structured suggested several independent introductions onto the various sampled islands and that these organisms subsequently evolved independently.

The *Continental cluster* contained multiple isolates from Vietnam (a primary sampling site), with three distinct subclusters, which captured the circulating lineages, *Vietnam 1*, *Vietnam 2* and *Vietnam Antimicrobial resistant (AMR)*. The Vietnam AMR subcluster was distinct from the other *S*. Weltevreden because 3/13 (23%) isolates had coding sequences that were associated with AMR (S1 Table), which overall we found to be rare within *S*. Weltevreden. Notably, with the overall phylogenetic structure of *S*. Weltevreden no particularly evident clustering was observed between the sources (environmental/human) of the bacterial isolates. Indeed, isolates from animal, food, the environment and humans were arbitrarily distributed throughout the tree. Furthermore, no evidence of significant temporal clustering was observed within the structure. Plasmid DNA was found to be common, with equivalents of pCM101 (the plasmid described in *S*. Weltevreden 10259) detectable in 90% of the sequenced isolates. A phylogenetic tree of the pCM101 structure mirrored that of the main chromosome, indicating that this plasmid has co-evolved with the chromosome, indicating a long-term likely synergistic relationship. This phylogenetic signal was determined by relatively little variation, with only 970 SNPs discriminating the plasmids on the phylogenetic tree. Further, when a single region of recombination was excluded from seven organisms that were isolated on La Réunion Island, the number of SNPs was reduced to only 48.

Many isolates scattered across the tree harbored more than one plasmid. Known AMR genes were detected in seven isolates. Isolate 2013_2776, originating from a food source in France (potentially imported from Southeast Asia) was found to harbor six AMR genes (*aph(3’)-Ia;* aminoglycoisides, *oqxA*/*B*; general efflux, *strA/B;* streptomycin and *tetB;* tetracycline, which were located on a plasmid similar to plasmid pSH111_227 (accession JN983042) found in *S*. Heidelberg. Indeed, of the isolates with laboratory confirmed resistance against antimicrobials (S1 Table), seven harbored additional predicted plasmids, equivalents of which have been previously associated with AMR (Table 2) [44–46].

**Table 2:** Summary of antimicrobial resistance genes identified in *Salmonella* Weltevreden and their nearest plasmid matches.

Gene	Antimicrobial resistance	Accession number of gene	Strain and plasmid of nearest match in Genbank
*qnrD*	Quinolones	FJ228229	**10347**
			*Klebsiella oxytoca* CAV1099 pKPC_CAV1099
*tetA*	Tetracyclines	AJ517790	**38_NTMD**
*qnrS1*	Quinolones	AB187515	*E*. *coli* strain 09/22a plasmid pEBG1
*bla*_*TEM30*_	Beta-lactams	AJ437107	**2011_02279**
			*E*. *coli* HUSEC2011 plasmid pHUSEC2011-1
*dfrA1*	Trimethoprim	JQ690541	**2013_2776**
*sul3*	Sulfonamides	AJ459418	*S*. Heidelberg plasmid pSH111_227
*aph3*	Aminoglycosides (gentamycin)	V00359	**2013_2776**
*oqxA*	Quinoxaline-di-*N*-oxide olaquindox	EU370913	*S*. Heidelberg plasmid pSH111_227
*oqxB*	Quinoxaline-di-*N*-oxide olaquindox	EU370913	
*strA*	Aminoglycosides (streptomycin)	NC_003384	
*strB*	Aminoglycosides (streptomycin)	M96392	
*tetB*	Tetracyclines	AF326777	

### The accessory genome content of *Salmonella* Weltevreden

A predicted pan genome of the sequenced *S*. Weltevreden isolates was created using annotated *de novo* assemblies. We identified a core of 4,046 CDSs present in each of the sequenced isolates using this approach, compared to 2,572 core CDSs identified using the broader set of *Salmonella* serovars. The total *S*. Weltevreden accessory genome was comprised of an additional 7,923 CDSs. An mean of 15 new predicted CDSs was added to the pan genome with every sequenced isolate and there were underlying collection of unique genes that were only found in single isolates (S2 Fig); the majority of these appeared to be attributable to mobile genetic elements and/or gene islands. Therefore, given the rate of novel gene acquisition and the increasing size of the pan genome, *S*. Weltevreden seems to have a submissive genome structure with the ability to routinely acquire and lose additional genetic material. Prophage-like regions are commonly associated with rapid evolution in *S*. *enterica* [47,48]. In order to align the phylogeny with phage-like signatures the genome sequences of 10259, C2346, 98_11262 and 99_3134, representing a cross section of diversity within the tree, were investigated using “PHAST” (http://phast.wishartlab.com/). Several apparently complete prophage sequences were identified within each bacterial isolate, with a mean of 12 prophage elements per isolate. Most prophages identified using PHAST were shared by all isolates; these included the classical *Salmonella* phages Gifsy 1, Gifsy 2, Fels 1 and entero PsP3 [49].

### The ability of *Salmonella* Weltevreden to invade cultured epithelial cells

**Fig 4:**
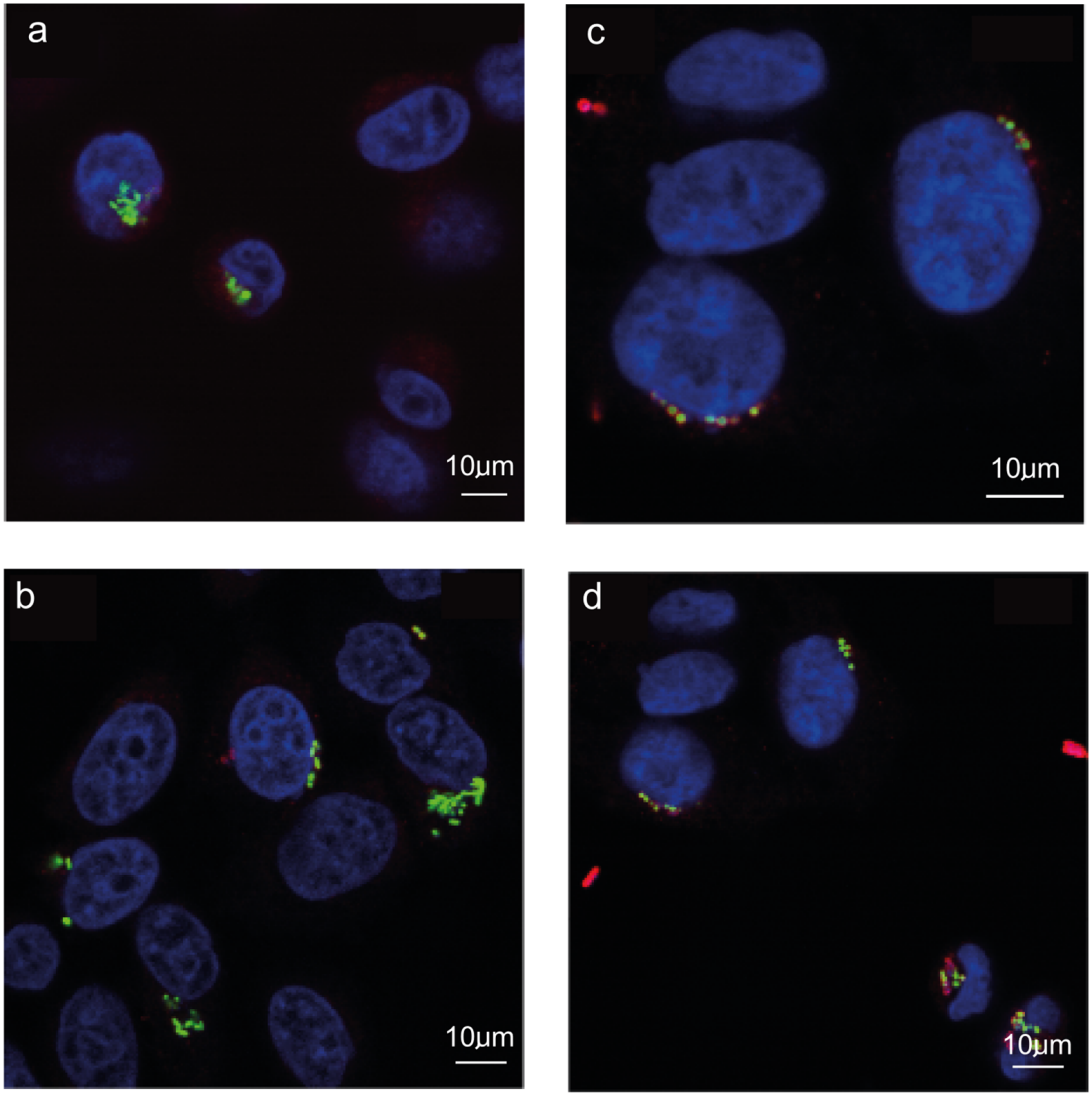
Confocal fluorescence microscopy image of S. Typhimurium SL1344 and S. Weltevreden in epithelial cells. Image shows S. Typhimurium SL1344 (a; two hours and b; six hours) and S. Weltevreden (c; two hours and d; six hours) interacting with Hep-2 cells at two and six hours post exposure. Cell nuclei are stained with DAPI (blue), common surface antigens (CSA) on extracellular Salmonella bacteria are stained in red and the Salmonella (pSsaG) where GFP expressing and are visible in green (GFP).

The *Salmonellae* have the ability to both adhere to and invade cultured cells [50]. Consequently, cultured Hep-2 cells were exposed independently to *S*. Typhimurium SL1344 (pSsaG), *S*. Weltevreden C2346(pSsaG) (Human asymptomatic stool isolate), *S*. Weltevreden 10259(pSsaG) Human symptomatic stool isolate), *S*. Weltevreden 98_11262(pSsaG) (Human bloodstream isolate) and *S*. Weltevreden 99_3134(pSsaG) (Human bloodstream isolate) at a multiplicity of infection (MOI) of ~50 bacteria per human cell (Fig 4). All *S*. Typhimurium and *S*. Weltevreden tested were able to invade Hep-2 epithelial cells. *S*. Typhimurium SL1344(pSsaG) exhibited a consistently stronger fluorescent signals at both two and six hours post infection, when compared to all *S*. Weltevreden. No significant difference in intracellular bacterial burden was observed between individual *S*. Weltevreden isolates. Importantly, there were consistently lower levels of GFP-positive *S*. Weltevreden using microscopic imaging than with *S*. Typhimurium, indicating that they are much less invasive in this assay than the archetypal *S*. Typhimurium SL1344.

**Fig 5:**
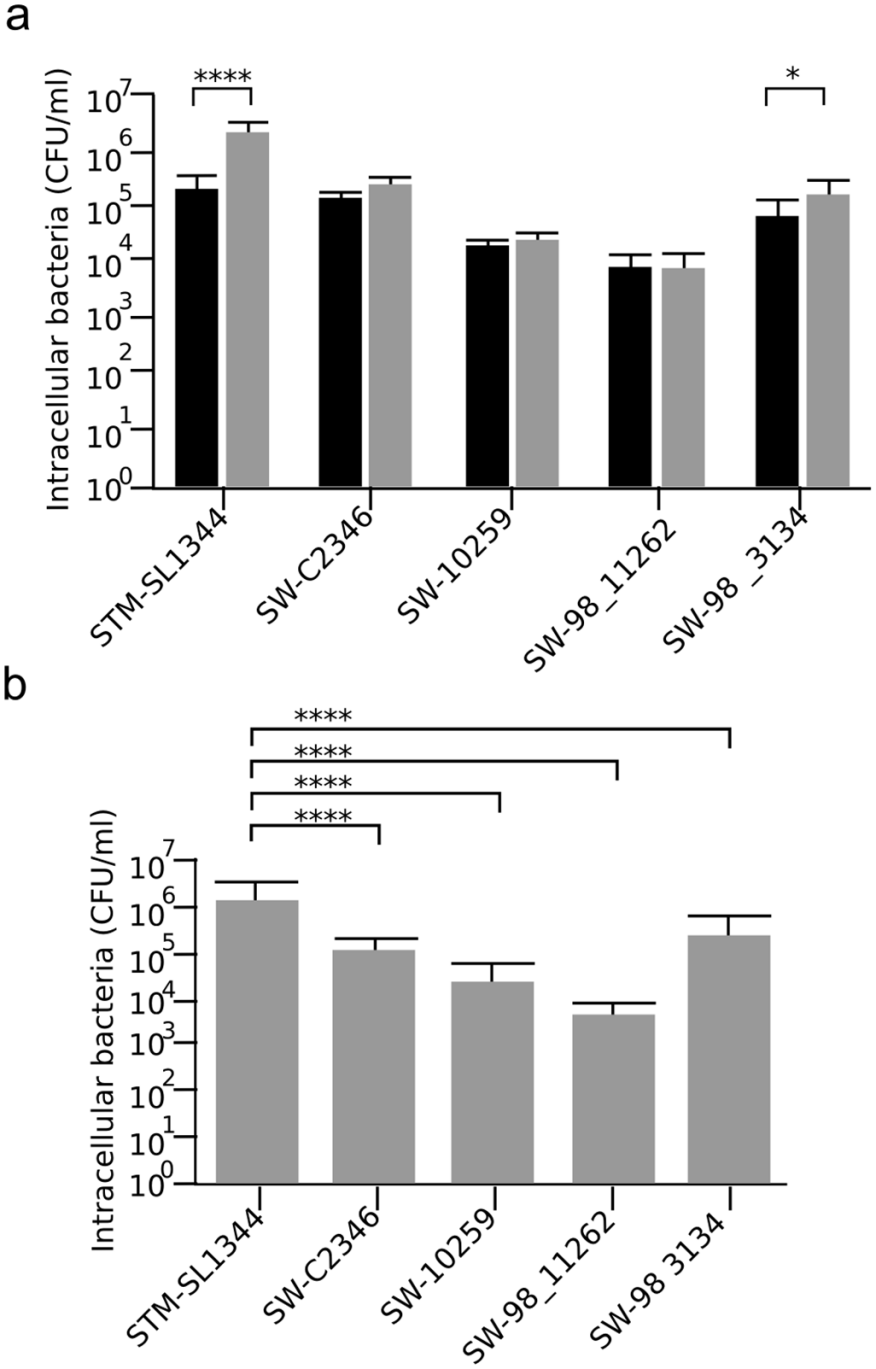
Viable Salmonella recovered in gentamicin killing assay with epithelial cells. a) Plots showing viable Salmonella (CFU/mL) recovered two (black) and six hours (grey) post infection after an invasion assay with Hep-2 cells infected with S. Typhimurium SL1344, S. Weltevreden SW C2346, SW 10259 SW98_11262 and SW99_3134 (MOI, 50); error bars indicate standard deviation. b) Plot of six hour time point only. Two-way ANOVA multiple comparisons were performed, significance is highlight by the symbols (* p<0.05, **** p<0.0001).

To assess the bacterial burden a gentamicin-killing assay was performed with infected Hep-2 epithelial cells. For *S*. Typhimurium SL1344, there was a consistent increase in the number of viable internalized bacteria between two and six hours post infection. At six hours post infection, there was a significant difference in the number of viable bacteria recovered compared to the two hour post infection time point for *S*. Typhimurium SL1344 (*p =* 0.0001, two sided t-test) (Fig 5). Conversely, no significant difference in recovered numbers was observed between the two and six hour time point for the *S*. Weltevreden isolates. Furthermore, there was a consistently lower level of invasion by all *S*. Weltevreden in comparison to *S*. Typhimurium SL1344.

### The potential virulence of *Salmonella* Weltevreden in a murine infection model

**Fig 6:**
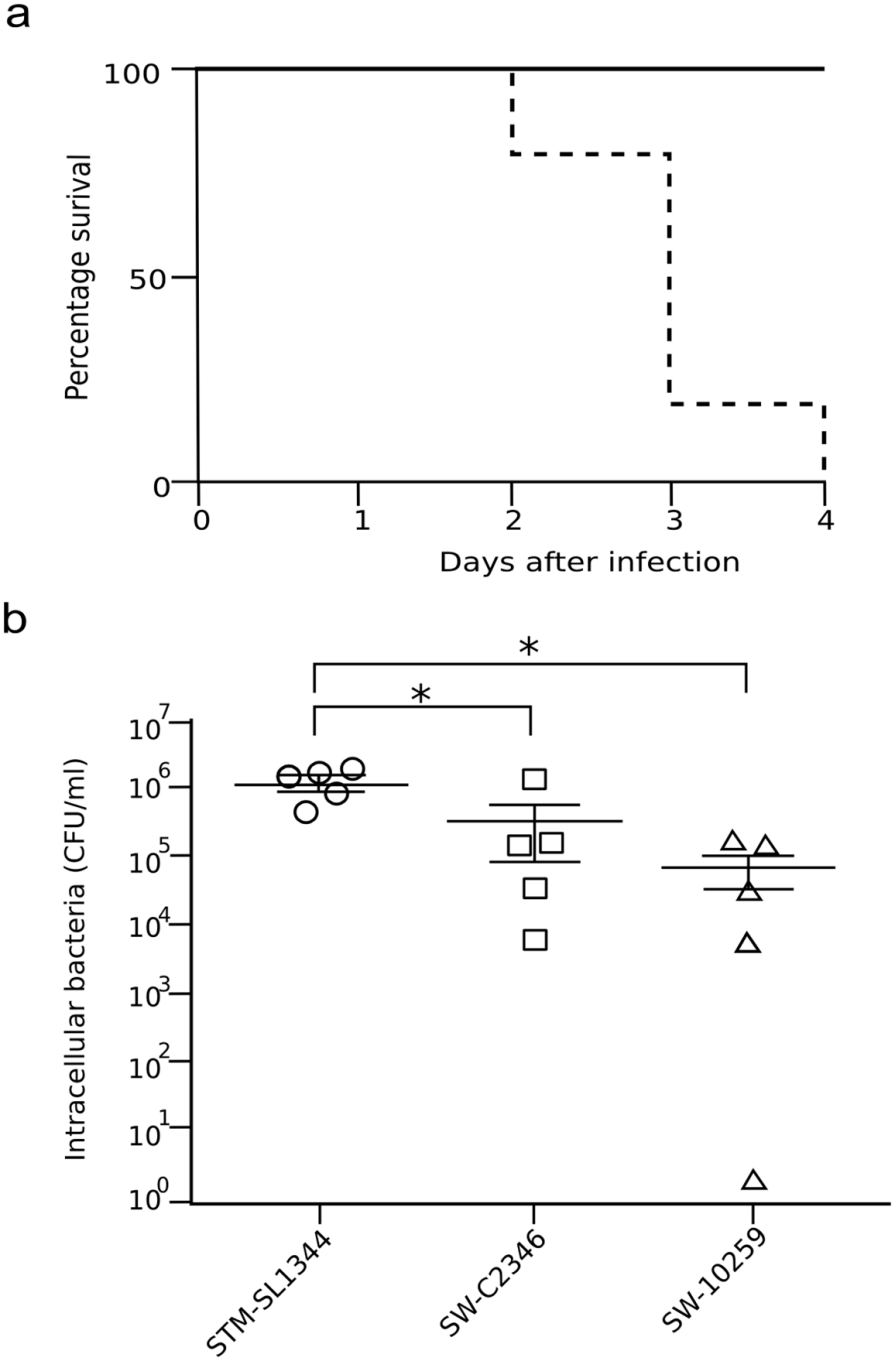
Murine challenge with S. Weltevreden in comparison to S. Typhimurium SL1344. a) Survival plot showing percentage survival of C57bl/6 mice challenged with S. Typhimurium SL1344, S. Weltevreden C2346 and S. Weltevreden 10259 following intravenous infection. Assays were performed in triplicate. b) Plots showing colonization of the murine caecum by S. Typhimurium SL1344, S. Weltevreden C2346 or S. Weltevreden 10259 in C57bl/6 mice at four days post infection. Differences in bacterial load were determined using the Mann Whitney Test identified (* p<0.05) Assays were performed in triplicate.

There are several murine models of *Salmonella* infection, which include the classical systemic typhoid model [51], and the streptomycin pre-treatment model [52], the latter of which is more relevant for gastroenteritis. Consequently, *S*. Weltevreden isolates were evaluated in both of these murine infection models in comparison again to *S*. Typhimurium SL1344. To determine the systemic virulence of *S*. Weltevreden, C57bl/6 (*Salmonella* susceptible, Nramp-1 negative) mice were infected intravenously with *S*. Typhimurium SL1344, *S*. Weltevreden C2346 and *S*. Weltevreden SW 10259 with 2,000 CFU. Mice were followed for four days to monitor disease severity. Mice infected with *S*. Weltevreden survived the four days post infection and remained well thereafter until being killed. In contrast, the majority of the C57bl/6 mice infected with *S*. Typhimurium SL1344 reached the disease severity endpoint two days post infection; others reached this state by day four and were killed (Fig 6).

In order to study the potential of *S*. Weltevreden to cause gastroenteritis, and to disaggregate the potential mechanism of infection, streptomycin pre-treated C57bl/6 mice were orally challenged with *S*. Weltevreden isolates C2346 and 10259 and *S*. Typhimurium SL1344; the inflammatory response and evidence of infection in the caecum were compared using histopathological analysis. Four days post infection, *S*. Weltevreden C2346 and 10259 induced pronounced inflammation on the caecum, characterized by edema in the submucosa, with distinct cellular infiltrate in the submucosa, the lamina propria, and the epithelial layer, as well as the presence of immune cells in the intestinal lumen. Crypt elongation and erosive changes in the surface epithelium were also observed. This inflammatory response was indistinguishable from *S*. Typhimurium SL1344. Thus, in contrast to the attenuated phenotype displayed by *S*. Weltevreden in the systemic murine model, a similar pattern in intestinal pathology were observed between both these serovars in the caecum after challenge of streptomycin-treated mice. The degree of colonization by the different *S*. *enterica* isolates was also measured by weighing sections of the caecum and liver and enumerating surviving *Salmonella* (Fig 6). *S*. Typhimurium SL1344 exhibited a significantly higher level of cecal and liver colonization in comparison to the *S*. Weltevreden isolates. In contrast, there was no significant difference in cecal and liver colonization between the *S*. Weltevreden isolates.

## Discussion

In this study, a combination of WGS, phylogenetic and *in-vitro*/*in-vivo* phenotyping were used to characterize the emerging *Salmonella* serovar *S*. Weltevreden. Additionally, four reference genomes (and corresponding reference strains) that will be of value for further genetic and genomic work on this increasing important serovar were generated. Our analysis revealed that the *S*. Weltevreden genome is larger than those of many other *S*. *enterica* serovars with a mean size over five million basepairs. Much of this additional genetic material was attributed to the accessory genome, where complete prophage and additional prophage-related elements were found to be common. Another little known serovar—*S*. Elisabethville, was found to be phylogenetically closest *S*. *enterica* serovar to *S*. Weltevreden. Remarkably, *S*. Elisabethville shares core serological properties with *S*. Weltevreden, but *S*. Elisabethville is not a common pathogen in humans. It will be interesting to see if this related serovar emerges in humans in the future, as has been the case for *S*. Weltevreden.

Our analysis additional found that *S*. Weltevreden is monophyletic serovar with two major phylogenetic clusters consisting of a largely “Continental” isolates and “Island” isolates. Thus, there is evidence of a significant degree of geographical structuring within the *S*. Weltevreden population. Some geographical clustering is also detectable within the subphylogeny, suggesting that *S*. Weltevreden continues to evolve within a specific geographical region, as opposed to frequently spreading from one location to another. Geographical subclustering has been detected in other serovars, including *S*. Typhimurium ST313 clades within sub-Saharan Africa [53]. These data suggest that after introduction *Salmonella* clades can become established in a specific environment or a human population where they can start to persist and evolve in an isolated manner. We found that 112 SNPs were cluster-specific; these could be of value for future epidemiological tracking studies. For example, it may be possible to determine if these SNPs can be used map potential transmission routes within and between different human and animal populations. Previous links have been described between seafood and *S*. Weltevreden causing human disease [54]. Here, some of the cluster-associated (or private SNPs) could be exploitable in SNP-based assays for the rapid identification of *S*. Weltevreden isolates in the field. Such approaches have been developed for other *Salmonella* serovars, including *S*. Typhi, for the urban and international tracking of typhoid fever [55,56].

It is meaningful that the phylogenetic structure of the *S*. Weltevreden sequenced here did not correlate with date of isolation, disease type or source (environment, animal, human). Therefore, it was not possible to link particular genotypes to disease syndromes. The inability to link genotype to human disease is suggestive, implying that factors such as infectious dose, host susceptibility and immune status or the local environment may be influencing the patterns of disease. More thorough epidemiological studies of *S*. Weltevreden are required to identify environment-to-human, animal-to-human, or human-to-human transmission routes. Notably, AMR was found not to play a big influence in shaping the molecular epidemiology of with *S*. Weltevreden, as AMR genes were found in very few isolates. The relatively rare AMR isolates harbored several plasmids comprised of structures that have been previously described in other Gram-negative bacteria. Thus, *S*. Weltevreden clearly has the capacity to acquire AMR genes, however they were rare. We speculate that the lack of AMR genes in this relatively submissive genome is related to the ecology of the organism, the it will be important to maintain surveillance on the serovar in order to actively detect any increasing AMR trends.

Phenotypic characterization of *S*. Weltevreden showed an overall attenuated virulence potential in different models of disease when compared to *S*. Typhimurium SL1344. *S*. Weltevreden isolates were significantly less invasive in terms of their ability to enter and replicate in Hep-2 cells. Similar to *in vitro* observations in Hep-2 cells, *S*. Weltevreden isolates were moderately attenuated in both the mouse in both intravenous and oral streptomycin treated infection models, than *S*. Typhimurium SL1344. In fact, mice intravenously infected with *S*. Weltevreden were able to survive four days post infection. However, despite this attenuation in Hep-2 cells and mice *S*. Weltevreden can clearly cause significant human disease and other approaches will be required to define virulence mechanisms associated with this emerging pathogen.

In conclusion, for the first time we have studied the phylogenetic structure and aimed to define the virulence potential of *S*. Weltevreden, an emerging cause of *Salmonella* induced infections in tropical regions. Our data show that *S*. Weltevreden is complex serovar and whilst we did observe geographical clustering this pathogen appears to have a more permeable genome that many other *Salmonella*. The acquisition of horizontally acquired DNA appears to be the main evolutionary driving force within the serovar, however AMR genes are rare. We additionally can show that *S*. Weltevreden has a distinct virulence-associated phenotype in conventional laboratory *Salmonella* virulence assays, which clearly suggests attenuation in comparison to *S*. Typhimurium SL1344. Our study provides new insights into this organism and will serve as a platform for future research on this emerging *Salmonella* serovar.

## Supporting Information

S1 Table The strains and sequence accession numbers of the *S*. Weltevreden included in this study. (DOCX)

S2 Table SNPs defining the Continental and Island clusters of *S*. Weltevreden. (DOCX)

S1 Fig Phylogenetic tress showing the population structure of S. Weltevreden isolates with recombination site. Red blocks on represent recombination events identified in comparison to the ancestral node and, blue blocks represent recombination events in a single isolate. Prophage regions and other areas of horizontal gene transfer are shown on the genome map.
(PDF)

S2 Fig The pan genome of *S*. Weltevreden. a) Plot showing variance in the number of unique genes found in a single isolate only and the number of new genes as genomes are added to the pan genome. b) Variance in the total number of predicted CDSs (genes) in the pan genome and the of conserved CDSs (99% of isolates) in the core genome as samples are added. c) Breakdown of the frequency of gene in isolates and in the overall collection of *S*. Weltevreden. Here, the core genome is defined by genes present in 99–100% of isolates, the soft-core by 95–99%, the shell by 15–95% and the cloud by 1–15%.
(PDF)
