# Supplementary material for: A Phylogenetic and Phenotypic Analysis of Salmonella enterica Serovar Weltevreden, an Emerging Agent of Diarrheal Disease in Tropical Regions
Source: PLoS Negl Trop Dis. 2016 Feb 11;10(2):e0004446. doi: 10.1371/journal.pntd.0004446 (PMC4750946; doi:10.1371/journal.pntd.0004446)
Supplement: S1 Table — (DOCX) [file pntd.0004446.s001.docx]

**S1 Table**. The strains and sequence accession numbers of the *S*. Weltevreden included in this study

| **Name** | **Year of isolation** | **Country** | **Host** | **Isolation source** | **Assembly Accessions** | **Sample Accession** | **Illumina Read Accession** | **PacBio Read Accession** |
| --- | --- | --- | --- | --- | --- | --- | --- | --- |
| 71_V_366 | Not known | Viet Nam | Duck | Duck | CZOW01000001-CZOW01000097 | ERS325044 | ERR387730 |  |
| 7_H_437 | Not known | Viet Nam | Pig | Pig | CZNW01000001-CZNW01000078 | ERS325045 | ERR387731 |  |
| 74_V_379 | Not known | Viet Nam | Duck | Duck | CZNR01000001-CZNR01000177 | ERS325046 | ERR387732 |  |
| SR134 | Not known | Viet Nam | Rat | Rat | CZOA01000001-CZOA01000080 | ERS325047 | ERR387733 |  |
| SR046 | Not known | Viet Nam | Rat | Rat | CZOM01000001-CZOM01000076 | ERS325048 | ERR387734 |  |
| 139K | 1940 | Indonesia | Homo sapiens | Human | CZNX01000001-CZNX01000071 | ERS394423 | ERR495205 |  |
| 840K | 1956 | Sri Lanka | Homo sapiens | Human | CZOX01000001-CZOX01000065 | ERS394424 | ERR495206 |  |
| 98_11262 | 1998 | New Caledonia | Homo sapiens | Human Stool | LN890522-LN890523 | ERS394425 | ERR495207 | ERR759467,ERR759470 |
| 99_3134 | 1999 | Reunion | Homo sapiens | Human Blood | LN890524-LN890526 | ERS394426 | ERR495208 | ERR759468,ERR759471 |
| 00_9824 | 2000 | Thailand | Homo sapiens | Human Stool | CZOV01000001-CZOV01000078 | ERS394427 | ERR495209 |  |
| 00_9879 | 2000 | Madagascar | Homo sapiens | Human Stool | CZON01000001-CZON01000063 | ERS394428 | ERR495210 |  |
| 02_1171 | 2002 | Thailand | Homo sapiens | Human Stool | CZPE01000001-CZPE01000074 | ERS394429 | ERR495211 |  |
| 03_1986 | 2003 | New Caledonia | Homo sapiens | Human Stool | CZNS01000001-CZNS01000062 | ERS394430 | ERR495212 |  |
| 03_4395 | 2003 | Guyana | Homo sapiens | Human Stool | CZOR01000001-CZOR01000072 | ERS394431 | ERR495213 |  |
| 03_5461 | 2003 | Guyana | Homo sapiens | Human Stool | CZOI01000001-CZOI01000069 | ERS394432 | ERR495214 |  |
| 05_0775 | 2005 | Mauritius | Homo sapiens | Human Stool | CZNV01000001-CZNV01000059 | ERS394433 | ERR495215 |  |
| 08_2437 | 2008 | Maldives | Homo sapiens | Human Stool | CZOY01000001-CZOY01000060 | ERS394434 | ERR495216 |  |
| 09_4703 | 2009 | Indonesia | Homo sapiens | Human Stool | CZOU01000001-CZOU01000072 | ERS394436 | ERR495218 |  |
| 09_5462 | 2009 | Guadeloupe | Homo sapiens | Human Stool | CZOJ01000001-CZOJ01000067 | ERS394437 | ERR495219 |  |
| 09_8500 | 2009 | Mauritius | Homo sapiens | Human Stool | CZPF01000001-CZPF01000052 | ERS394438 | ERR495220 |  |
| 201005280 | 2010 | Indonesia | Homo sapiens | Human Stool | CZOF01000001-CZOF01000088 | ERS394439 | ERR495221 |  |
| 201009500 | 2010 | Laos | Homo sapiens | Human Stool | CZNQ01000001-CZNQ01000083 | ERS394440 | ERR495222 |  |
| 201008825 | 2010 | Malaysia | Homo sapiens | Human Stool | CZOT01000001-CZOT01000067 | ERS394441 | ERR495223 |  |
| 201007622 | 2010 | Sri Lanka | Homo sapiens | Human Stool | CZNU01000001-CZNU01000050 | ERS394442 | ERR495224 |  |
| 201008132 | 2010 | India | Homo sapiens | Human Stool | CZOH01000001-CZOH01000057 | ERS394443 | ERR495225 |  |
| 201009035 | 2010 | Mayotte | Homo sapiens | Human Stool | CZNO01000001-CZNO01000055 | ERS394444 | ERR495226 |  |
| 201008341 | 2010 | French Polynesia: Tahiti | Homo sapiens | Human Stool | CZPP01000001-CZPP01000049 | ERS394445 | ERR495227 |  |
| 201006598 | 2010 | Reunion | Homo sapiens | Human Stool | CZOB01000001-CZOB01000050 | ERS394446 | ERR495228 |  |
| 201111351 | 2011 | Guadeloupe | Homo sapiens | Human Stool | CZOD01000001-CZOD01000079 | ERS394455 | ERR495237 |  |
| 201205005 | 2012 | French Polynesia: Tahiti | Homo sapiens | Human Stool | CZPM01000001-CZPM01000047 | ERS394456 | ERR495238 |  |
| 201201335 | 2012 | French Polynesia: Tahiti | Homo sapiens | Human Stool | CZNY01000001-CZNY01000050 | ERS394457 | ERR495239 |  |
| 201208537 | 2012 | New Caledonia | Homo sapiens | Human Stool | CZNT01000001-CZNT01000055 | ERS394458 | ERR495240 |  |
| 201202227 | 2012 | Reunion | Homo sapiens | Human Stool | CZPB01000001-CZPB01000058 | ERS394459 | ERR495241 |  |
| 201201497 | 2012 | Thailand | Homo sapiens | Human Stool | CZOG01000001-CZOG01000076 | ERS394460 | ERR495242 |  |
| 201202882 | 2012 | Reunion | Homo sapiens | Human Stool | CZPD01000001-CZPD01000056 | ERS394461 | ERR495243 |  |
| 201201076 | 2012 | Reunion | Homo sapiens | Human Stool | CZPC01000001-CZPC01000058 | ERS394462 | ERR495244 |  |
| 201300011 | 2013 | Reunion | Homo sapiens | Human Stool | CZOO01000001-CZOO01000071 | ERS394463 | ERR495245 |  |
| 201300482 | 2013 | French Polynesia: Tahiti | Homo sapiens | Human Stool | CZPH01000001-CZPH01000050 | ERS394464 | ERR495246 |  |
| 201100200 | 2011 | Reunion | Homo sapiens | Human Stool | CZNZ01000001-CZNZ01000054 | ERS394447 | ERR495229 |  |
| 201302134 | 2013 | French Polynesia: Tahiti | Homo sapiens | Human Stool | CZPO01000001-CZPO01000048 | ERS394465 | ERR495247 |  |
| 201302479 | 2013 | Algeria | Homo sapiens | Human Stool | CZOL01000001-CZOL01000083 | ERS394466 | ERR495248 |  |
| 201303357 | 2013 | Thailand | Homo sapiens | Human Stool | CZOP01000001-CZOP01000069 | ERS394467 | ERR495249 |  |
| 201304810 | 2013 | New Caledonia | Homo sapiens | Human Stool | CZPA01000001-CZPA01000057 | ERS394468 | ERR495250 |  |
| 201304851 | 2013 | Guyana | Homo sapiens | Human Stool | CZOC01000001-CZOC01000081 | ERS394469 | ERR495251 |  |
| 201305036 | 2013 | Reunion | Homo sapiens | Human Stool | CZNP01000001-CZNP01000059 | ERS394470 | ERR495252 |  |
| 201305421 | 2013 | Thailand | Homo sapiens | Human Stool | CZOK01000001-CZOK01000067 | ERS394471 | ERR495253 |  |
| 2006_3740 | 2006 | France | Fish | Fish | CZOQ01000001-CZOQ01000073 | ERS394472 | ERR495254 |  |
| 2006_3866 | 2006 | France | Seafood | Seafood | CZPG01000001-CZPG01000076 | ERS394473 | ERR495255 |  |
| 2013_3452 | 2013 | Reunion | Food | Food | CZOZ01000001-CZOZ01000055 | ERS394474 | ERR495256 |  |
| 201100324 | 2011 | French Polynesia: Tahiti | Homo sapiens | Human Stool | CZOS01000001-CZOS01000052 | ERS394448 | ERR495230 |  |
| 2013_3736 | 2013 | Reunion | Poultry | Poultry | CZPN01000001-CZPN01000048 | ERS394475 | ERR495257 |  |
| 2013_3101 | 2013 | New Caledonia | Food | Food | CZOE01000001-CZOE01000058 | ERS394476 | ERR495258 |  |
| 2013_3456 | 2013 | Reunion | Food | Food | CZPQ01000001-CZPQ01000047 | ERS394477 | ERR495259 |  |
| 2013_3102 | 2013 | New Caledonia | Food | Food | CZPI01000001-CZPI01000064 | ERS394478 | ERR495260 |  |
| 2013_3667 | 2013 | Reunion | Poultry | Poultry | CZMJ01000001-CZMJ01000056 | ERS394479 | ERR495261 |  |
| 2013_4066 | 2013 | France | Seafood | Seafood | CZLP01000001-CZLP01000056 | ERS394480 | ERR495262 |  |
| 2012_2474 | 2012 | Reunion | Poultry | Poultry | CZLI01000001-CZLI01000058 | ERS394481 | ERR495263 |  |
| 2012_2981 | 2012 | Reunion | Environment feeding stuffs plant | Environment feeding stuffs plant | CZMK01000001-CZMK01000059 | ERS394482 | ERR495264 |  |
| 2012_3395 | 2012 | India | Seafood | Seafood | CZPK01000001-CZPK01000044 | ERS394483 | ERR495265 |  |
| 2012_3614 | 2012 | India | Seafood | Seafood | CZLZ01000001-CZLZ01000069 | ERS394484 | ERR495266 |  |
| 201100503 | 2011 | Guadeloupe | Homo sapiens | Human Stool | CZNB01000001-CZNB01000069 | ERS394449 | ERR495231 |  |
| 2012_4538 | 2012 | Reunion | Food | Food | CZMH01000001-CZMH01000058 | ERS394485 | ERR495267 |  |
| 2012_4716 | 2012 | Reunion | Environment | Environment | CZLJ01000001-CZLJ01000059 | ERS394486 | ERR495268 |  |
| 2013_2515 | 2013 | India | Poultry | Poultry | CZPL01000001-CZPL01000047 | ERS394487 | ERR495269 |  |
| 2013_2518 | 2013 | India | Environment | Environment | CZMW01000001-CZMW01000060 | ERS394488 | ERR495270 |  |
| 2013_2776 | 2013 | France | Vegetable | Vegetable | CZMI01000001-CZMI01000079 | ERS394489 | ERR495271 |  |
| 2013_2778 | 2013 | New Caledonia | Seafood | Seafood | CZLT01000001-CZLT01000058 | ERS394490 | ERR495272 |  |
| 2013_2847 | 2013 | Reunion | Poultry | Poultry | CZLK01000001-CZLK01000056 | ERS394491 | ERR495273 |  |
| 2013_2908 | 2013 | New Caledonia | Porcine Feed | Porcine Feed | CZMB01000001-CZMB01000052 | ERS394492 | ERR495274 |  |
| 2013_2912 | 2013 | New Caledonia | Porcine Feed | Porcine Feed | CZMS01000001-CZMS01000053 | ERS394493 | ERR495275 |  |
| 2013_69 | 2013 | New Caledonia | Bovine | Bovine | CZLR01000001-CZLR01000056 | ERS394494 | ERR495276 |  |
| 201100823 | 2011 | Indonesia | Homo sapiens | Human Stool | CZMY01000001-CZMY01000075 | ERS394450 | ERR495232 |  |
| 2013_71 | 2013 | France | Vegetable | Vegetable | CZLM01000001-CZLM01000072 | ERS394495 | ERR495277 |  |
| 2013_101 | 2013 | France | Seafood | Seafood | CZMX01000001-CZMX01000076 | ERS394496 | ERR495278 |  |
| 2013_690 | 2013 | not applicable | Seafood | Seafood | CZMN01000001-CZMN01000051 | ERS394497 | ERR495279 |  |
| 2013_1005 | 2013 | France | Pork | Pork | CZMP01000001-CZMP01000083 | ERS394498 | ERR495280 |  |
| 2013_1032 | 2013 | France | Pork | Pork | CZML01000001-CZML01000087 | ERS394499 | ERR495281 |  |
| 2013_1051 | 2013 | France | Poultry | Poultry | CZLW01000001-CZLW01000050 | ERS394500 | ERR495282 |  |
| 2013_1418 | 2013 | France | Fish | Fish | CZMU01000001-CZMU01000079 | ERS394501 | ERR495283 |  |
| 2013_1467 | 2013 | France | Vegetable | Vegetable | CZMC01000001-CZMC01000055 | ERS394502 | ERR495284 |  |
| 201102279 | 2011 | Mayotte | Homo sapiens | Human Stool | CZLO01000001-CZLO01000066 | ERS394451 | ERR495233 |  |
| 201103604 | 2011 | French Polynesia: Tahiti | Homo sapiens | Human Stool | CZMA01000001-CZMA01000057 | ERS394452 | ERR495234 |  |
| 201107037 | 2011 | Guyana | Homo sapiens | Human Stool | CZMG01000001-CZMG01000060 | ERS394453 | ERR495235 |  |
| 201109395 | 2011 | India | Homo sapiens | Human Stool | CZMZ01000001-CZMZ01000087 | ERS394454 | ERR495236 |  |
| VNB635 | 2009 | Viet Nam | Homo sapiens | Human Blood | CZLX01000001-CZLX01000066 | ERS208983 | ERR294781 |  |
| VNS30438 | 2010 | Viet Nam | Homo sapiens | Human Stool | CZMR01000001-CZMR01000080 | ERS208992 | ERR294790 |  |
| VNS72_LNPT | 2007 | Viet Nam | Homo sapiens | Human Stool | CZMD01000001-CZMD01000076 | ERS208993 | ERR294791 |  |
| VNS63_AYSA | 2007 | Viet Nam | Homo sapiens | Human Stool | CZLQ01000001-CZLQ01000081 | ERS208994 | ERR294792 |  |
| VNS106_MR | 2007 | Viet Nam | Homo sapiens | Human Stool | CZMF01000001-CZMF01000086 | ERS208995 | ERR294793 |  |
| VNS94_VNQN | 2007 | Viet Nam | Homo sapiens | Human Stool | CZLU01000001-CZLU01000074 | ERS208996 | ERR294794 |  |
| VNS132_NTNK | 2007 | Viet Nam | Homo sapiens | Human Stool | CZMM01000001-CZMM01000078 | ERS208997 | ERR294795 |  |
| VNS8_LTPO | 2007 | Viet Nam | Homo sapiens | Human Stool | CZLY01000001-CZLY01000074 | ERS208998 | ERR294796 |  |
| VNS38_NTMD | 2007 | Viet Nam | Homo sapiens | Human Stool | CZMO01000001-CZMO01000074 | ERS208999 | ERR294797 |  |
| VNS46_DNBH | 2007 | Viet Nam | Homo sapiens | Human Stool | CZLV01000001-CZLV01000092 | ERS209000 | ERR294798 |  |
| VNS184_VTHT | 2007 | Viet Nam | Homo sapiens | Human Stool | CZMQ01000001-CZMQ01000054 | ERS209001 | ERR294799 |  |
| VNS10162 | 2009 | Viet Nam | Homo sapiens | Human Stool | CZLS01000001-CZLS01000070 | ERS208984 | ERR294782 |  |
| VNS194_SL | 2007 | Viet Nam | Homo sapiens | Human Stool | CZNC01000001-CZNC01000085 | ERS209002 | ERR294800 |  |
| VNS179_LTKT | 2007 | Viet Nam | Homo sapiens | Human Stool | CZLL01000001-CZLL01000086 | ERS209003 | ERR294801 |  |
| VNS008_PNTL | 2007 | Viet Nam | Homo sapiens | Human Stool | CZNA01000001-CZNA01000075 | ERS209004 | ERR294802 |  |
| VNS170_NVTN | 2007 | Viet Nam | Homo sapiens | Human Stool | CZME01000001-CZME01000087 | ERS209005 | ERR294803 |  |
| VNSC2142 | 2010 | Viet Nam | Homo sapiens | Human Stool | CZMT01000001-CZMT01000092 | ERS209006 | ERR294804 |  |
| VNSC2036 | 2010 | Viet Nam | Homo sapiens | Human Stool | CZLN01000001-CZLN01000070 | ERS209007 | ERR294805 |  |
| VNSC2248 | 2010 | Viet Nam | Homo sapiens | Human Stool | CZMV01000001-CZMV01000072 | ERS209009 | ERR294807 |  |
| VNSC2346 | 2010 | Viet Nam | Homo sapiens | Human Stool | LN890520-LN890521 | ERS209010 | ERR294808 | ERR1079226 |
| VNS10259 | 2009 | Viet Nam | Homo sapiens | Human Stool | LN890518-LN890519 | ERS208985 | ERR294783 | ERR1079227 |
| VNSC2377 | 2010 | Viet Nam | Homo sapiens | Human Stool | CZNE01000001-CZNE01000073 | ERS209012 | ERR294810 |  |
| VNSC2471 | 2010 | Viet Nam | Homo sapiens | Human Stool | CZND01000001-CZND01000065 | ERS209013 | ERR294811 |  |
| VNSC2511 | 2010 | Viet Nam | Homo sapiens | Human Stool | CZNF01000001-CZNF01000101 | ERS209014 | ERR294812 |  |
| VNSC2512 | 2010 | Viet Nam | Homo sapiens | Human Stool | CZNG01000001-CZNG01000069 | ERS209015 | ERR294813 |  |
| VNS003_D14 | 2005 | Viet Nam | Homo sapiens | Human Stool | CZNM01000001-CZNM01000079 | ERS209016 | ERR294814 |  |
| VNS10290 | 2009 | Viet Nam | Homo sapiens | Human Stool | CZNL01000001-CZNL01000073 | ERS208986 | ERR294784 |  |
| VNS10347 | 2009 | Viet Nam | Homo sapiens | Human Stool | CZNH01000001-CZNH01000075 | ERS208987 | ERR294785 |  |
| VNS20069 | 2009 | Viet Nam | Homo sapiens | Human Stool | CZNK01000001-CZNK01000067 | ERS208988 | ERR294786 |  |
| VNS20372 | 2009 | Viet Nam | Homo sapiens | Human Stool | CZNN01000001-CZNN01000070 | ERS208989 | ERR294787 |  |
| VNS20510 | 2009 | Viet Nam | Homo sapiens | Human Stool | CZNI01000001-CZNI01000077 | ERS208990 | ERR294788 |  |
| VNS30291 | 2009 | Viet Nam | Homo sapiens | Human Stool | CZNJ01000001-CZNJ01000075 | ERS208991 | ERR294789 |  |
