# Supplementary material for: A Phylogenetic and Phenotypic Analysis of Salmonella enterica Serovar Weltevreden, an Emerging Agent of Diarrheal Disease in Tropical Regions
Source: PLoS Negl Trop Dis. 2016 Feb 11;10(2):e0004446. doi: 10.1371/journal.pntd.0004446 (PMC4750946; doi:10.1371/journal.pntd.0004446)
Supplement: S2 Table — (DOCX) [file pntd.0004446.s002.docx]

**S2 Table**. SNPs defining the Continental and Island clusters of *S*. Weltevreden

| **Genome Coordinates** | **Continental SNP** | **Islands SNP** | **SNP Type** | **Amino acid change** | **Gene name** | **Gene function** |
| --- | --- | --- | --- | --- | --- | --- |
| 38110 | T | G | Nonsynonymous | M1I | yhcR | secreted 5'-nucleotidase |
| 53518 | C | G | Nonsynonymous | V1M | ribF | riboflavin biosynthesis protein RibF |
| 150218 | T | C | Synonymous | 287R | murD | UDP-N-acetylmuramoyl-L-alanine:D-glutamate ligase |
| 159866 | G | A | Intergenic |  |  |  |
| 165171 | A | G | Synonymous | 48V | lysR_1 | LysR family transcriptional regulator |
| 171185 | G | C | Nonsynonymous | Y110H | hofB | protein transport protein HofB |
| 186542 | A | G | Intergenic |  |  |  |
| 455296 | A | G | Intergenic |  |  |  |
| 482321 | T | C | Nonsynonymous | T116A | yajI | lipoprotein |
| 493751 | G | A | Intergenic |  |  |  |
| 551959 | T | A | Nonsynonymous | D132N | ybaN | Inner membrane protein YbaN |
| 553182 | G | T | Intergenic |  |  |  |
| 555948 | G | A | Nonsynonymous | R136C | SBOV4431 | chaperone protein HtpG |
| 623261 | A | G | Intergenic |  |  |  |
| 666014 | A | G | Intergenic |  |  |  |
| 667137 | A | G | Intergenic |  |  |  |
| 694039 | C | A | Nonsynonymous | A14V | entF | enterobactin synthetase component F |
| 731339 | G | A | Nonsynonymous | N142K | citF_2 | citrate lyase subunit alpha |
| 877723 | A | G | Nonsynonymous | L16P | sdcS_1 | cation transporter |
| 882274 | C | T | Nonsynonymous | N160T | 10259_00846 | membrane protein |
| 885476 | T | C | Synonymous | 245N | gpmA | phosphoglyceromutase |
| 928510 | C | T | Synonymous | 217L | ybhL_1 | membrane protein |
| 969008 | A | G | Intergenic |  |  |  |
| 1087552 | T | C | Synonymous | 94D | cydD | cysteine/glutathione ABC transporter membrane/ATP-binding component |
| 1102354 | C | T | Nonsynonymous | L171F | dmsB_2 | anaerobic dimethyl sulfoxide reductase subunit B |
| 1151458 | C | T | Synonymous | 268T | 10259_01111 | amino acid:proton symporter |
| 1202670 | T | A | Nonsynonymous | S177P | pepN | aminopeptidase N |
| 1283933 | T | C | Synonymous | 70P | 10259_01257 | hypothetical protein |
| 1461980 | A | T | Intergenic |  |  |  |
| 1539870 | A | G | Intergenic |  |  |  |
| 1560429 | T | C | Intergenic |  |  |  |
| 1625658 | G | A | Intergenic |  |  |  |
| 1669118 | G | A | Intergenic |  |  |  |
| 1694992 | A | C | Intergenic |  |  |  |
| 1697445 | G | A | Nonsynonymous | P186L | SBOV16411 | putative inner membrane protein |
| 1703784 | G | C | Nonsynonymous | L19R | 10259_01695 | protein ydcJ |
| 1720617 | T | C | Synonymous | 76N | gatC_1 | phosphotransferase enzyme |
| 1735902 | C | A | Nonsynonymous | V190I | ydcR_1 | GntR family transcriptional regulator |
| 1743929 | A | G | Synonymous | 37R | 10259_01734 | ssrAB activated gene |
| 1782320 | G | A | Intergenic |  |  |  |
| 1792507 | C | T | Synonymous | 243Y | galS_1 | transcriptional regulator |
| 1827483 | T | G | Intergenic |  |  |  |
| 1842679 | C | T | Nonsynonymous | P191L | 10259_01827 | lipoprotein |
| 1904277 | G | A | Synonymous | 33P | ydhJ | multidrug resistance efflux pump |
| 1938526 | G | T | Nonsynonymous | Y191H | sseC | pathogenicity island 2 effector protein SseC |
| 1945318 | G | C | Nonsynonymous | A206T | ssrA | sensor kinase |
| 1948148 | C | T | Nonsynonymous | P212A | ycgE_1 | MerR family transcriptional regulator |
| 1952986 | T | C | Nonsynonymous | N234H | ttrB | tetrathionate reductase subunit B |
| 1955378 | C | T | Synonymous | 391L | ttrA | tetrathionate reductase subunit A |
| 2072991 | C | T | Synonymous | 395H | dosC | diguanylate cylase |
| 2104434 | A | T | Intergenic |  |  |  |
| 2155215 | T | C | Synonymous | 29G | mnmA | tRNA-specific 2-thiouridylase MnmA |
| 2179555 | A | C | Nonsynonymous | I24T | ycfS | LD-transpeptidase YcfS |
| 2306947 | T | G | Nonsynonymous | I253V | ackA_1 | propionate kinase |
| 2312418 | C | T | Nonsynonymous | V262L | dacD | penicillin-binding protein |
| 2327448 | G | A | Synonymous | 20L | hisC | histidinol-phosphate aminotransferase |
| 2349279 | C | T | Nonsynonymous | G266D | rfbD_2 | dTDP-4-dehydrorhamnose reductase |
| 2368620 | T | C | Nonsynonymous | P271H | wcaC | glycosyltransferase |
| 2388569 | G | A | Nonsynonymous | G3R | yegN | RND family transporter protein |
| 2397768 | A | G | Intergenic |  |  |  |
| 2487266 | C | T | Nonsynonymous | N31H | yeiO | sugar efflux transporter |
| 2571886 | C | A | Synonymous | 233G | arnB | UDP-4-amino-4-deoxy-L-arabinose--oxoglutarate aminotransferase |
| 2644752 | C | T | Nonsynonymous | A315V | folC | folylpolyglutamate synthase |
| 2655437 | C | A | Intergenic |  |  |  |
| 2745065 | A | G | Synonymous | 152R | xapA | purine nucleoside phosphorylase |
| 2751842 | T | C | Intergenic |  |  |  |
| 2806294 | A | C | Nonsynonymous | T319I | dapE | succinyl-diaminopimelate desuccinylase |
| 2830342 | A | G | Nonsynonymous | I331S | ppx | exopolyphosphatase |
| 2918022 | A | C | Nonsynonymous | H332D | dmsA_4 | putative anaerobic dimethylsulfoxide reductase |
| 2939599 | T | C | Nonsynonymous | L349Q | 10259_02939 | reductase |
| 3029090 | G | A | Nonsynonymous | L36P | nadB | L-aspartate oxidase |
| 3044150 | G | A | Synonymous | 372K | kgtP | alpha-ketoglutarate transporter |
| 3165103 | G | T | Nonsynonymous | G366S | gabR | DeoR family transcriptional regulator |
| 3192557 | G | A | Synonymous | 87L | srlA | Glucitol/sorbitol permease IIC component |
| 3247227 | A | G | Synonymous | 216Q | spaR | virulence associated secretory protein |
| 3250702 | G | A | Synonymous | 12Q | spaI | secretory apparatus ATP synthase (associated with virulence) |
| 3258960 | A | C | Intergenic |  |  |  |
| 3323082 | G | A | Nonsynonymous | W394C | SBOV30001 | conserved hypothetical protein |
| 3469564 | A | G | Synonymous | 84Q | yqgD | inner membrane protein |
| 3502405 | T | C | Nonsynonymous | R422L | 10259_03509 | FIC domain-containing protein |
| 3559237 | G | T | Nonsynonymous | E431Q | STY3343 | putative exported protein |
| 3638294 | T | C | Nonsynonymous | R451L | tdcD | propionate/acetate kinase |
| 3710463 | G | A | Synonymous | 122L | mtgA | monofunctional biosynthetic peptidoglycan transglycosylase |
| 3715222 | T | G | Intergenic |  |  |  |
| 3734518 | G | A | Intergenic |  |  |  |
| 3784257 | T | C | Synonymous | 260V | acrF | acriflavin resistance protein F |
| 3798695 | G | A | Synonymous | 51P | fmt | methionyl-tRNA formyltransferase |
| 3844549 | C | T | Intergenic |  |  |  |
| 3861654 | A | G | Synonymous | 68* | aroB | 3-dehydroquinate synthase |
| 3933453 | A | G | Intergenic |  |  |  |
| 3935103 | G | A | Synonymous | 226S | php | phosphotriesterase |
| 4022010 | T | A | Nonsynonymous | A49V | 10259_04023 | putative inner membrane protein |
| 4054900 | G | A | Nonsynonymous | D498G | xylR | xylose operon regulatory protein |
| 4087478 | C | A | Intergenic |  |  |  |
| 4151451 | A | G | Intergenic |  |  |  |
| 4179949 | T | C | Synonymous | 81N | 10259_04171 | putative secreted protein |
| 4183764 | C | A | Intergenic |  |  |  |
| 4202714 | G | A | Nonsynonymous | A51T | dsdX | permease |
| 4231325 | T | G | Nonsynonymous | M5118T | 10259_04223 | 2-oxo-3-deoxygalactonate kinase |
| 4343874 | A | G | Nonsynonymous | S55R | hemC | porphobilinogen deaminase |
| 4464873 | G | A | Nonsynonymous | L550Q | siaT_2 | integral membrane transport protein |
| 4469553 | A | C | Nonsynonymous | A561E | cpxA | two-component sensor kinase protein |
| 4478528 | G | A | Synonymous | 175K | yicJ_2 | sodium:galactoside symporter |
| 4525097 | G | A | Intergenic |  |  |  |
| 4528083 | T | C | Intergenic |  |  |  |
| 4537997 | A | G | Intergenic |  |  |  |
| 4613302 | A | G | Nonsynonymous | T68A | 10259_04597 | histidine biosynthesis protein |
| 4651837 | A | T | Intergenic |  |  |  |
| 4659202 | C | T | Synonymous | 224N | bepC | type-I secretion protein |
| 4676493 | T | C | Nonsynonymous | N697H | 10259_04641 | Ig domain-containing protein |
| 4740492 | T | C | Intergenic |  |  |  |
| 4783210 | C | T | Nonsynonymous | P7S | sugE | SugE protein |
| 4785518 | A | C | Nonsynonymous | H71Y | frdB | fumarate reductase, iron-sulfur protein |
| 4794271 | C | T | Nonsynonymous | S75T | psd | phosphatidylserine decarboxylase proenzyme |
| 4863047 | C | T | Intergenic |  |  |  |
| 4865401 | G | A | Nonsynonymous | F78I | iolB | 5-deoxyglucuronate isomerase |
| 4867756 | G | T | Intergenic |  |  |  |
| 4867800 | G | A | Intergenic |  |  |  |
| 4872126 | T | C | Synonymous | 359D | 10259_04826 | lysosomal glucosyl ceramidase |
| 4883973 | G | A | Synonymous | 37E | pmbA | peptidase PmbA |
| 4901727 | G | A | Nonsynonymous | G84D | mgtA | magnesium-transporting ATPase MgtA |
| 4982215 | C | G | Nonsynonymous | R88Q | mdtM | sugar transport protein |
