# Supplementary figures and images for: A Phylogenetic and Phenotypic Analysis of Salmonella enterica Serovar Weltevreden, an Emerging Agent of Diarrheal Disease in Tropical Regions
Source: PLoS Negl Trop Dis. 2016 Feb 11;10(2):e0004446. doi: 10.1371/journal.pntd.0004446 (PMC4750946; doi:10.1371/journal.pntd.0004446)

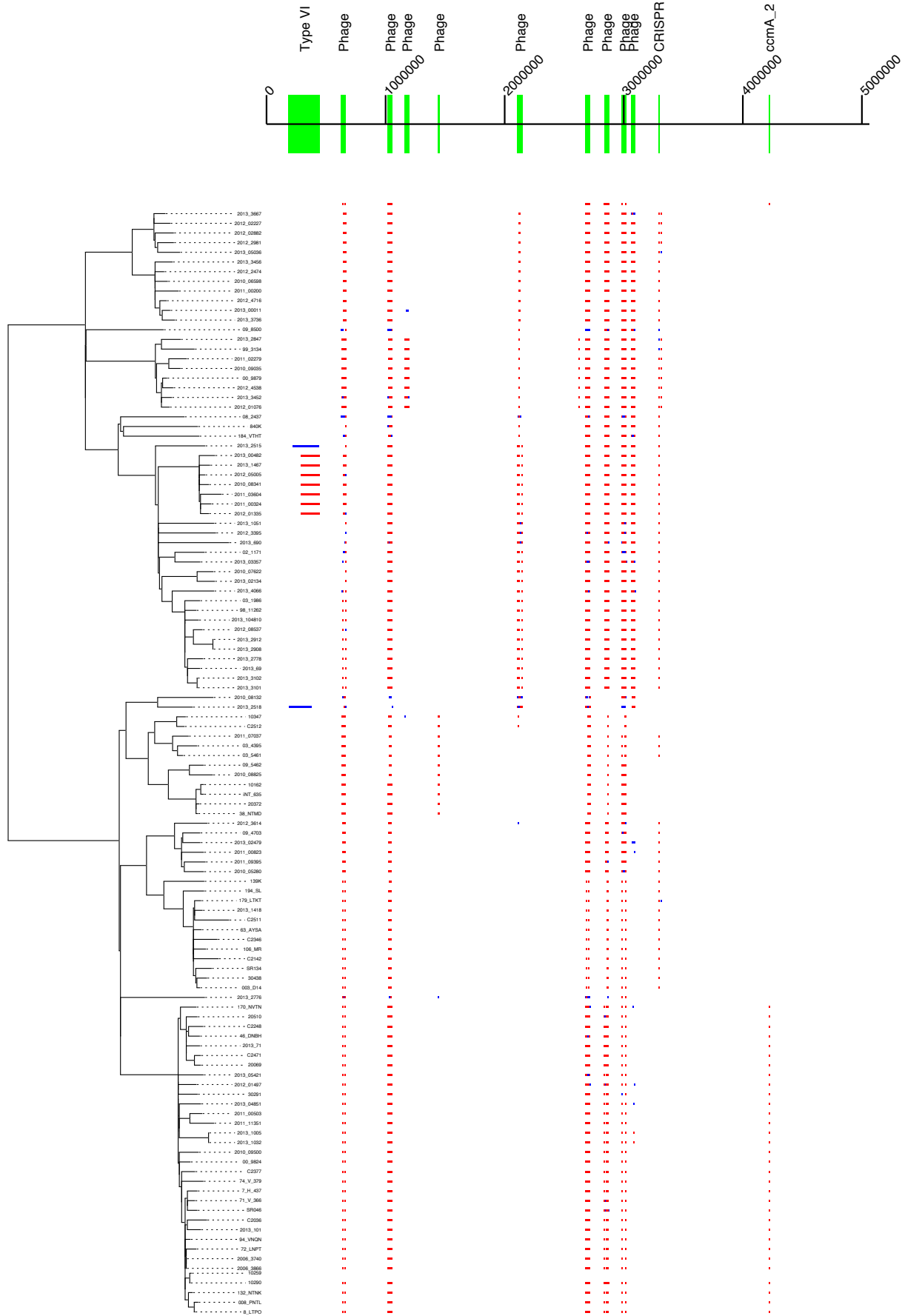

Supplement: S1 Fig — Red blocks on represent recombination events identified in comparison to the ancestral node and, blue blocks represent recombination events in a single isolate. Prophage regions and other areas of horizontal gene transfer are shown on the genome map. (PDF) [file pntd.0004446.s003.pdf]

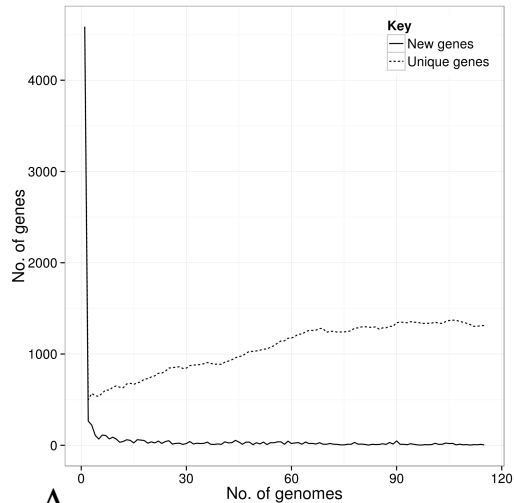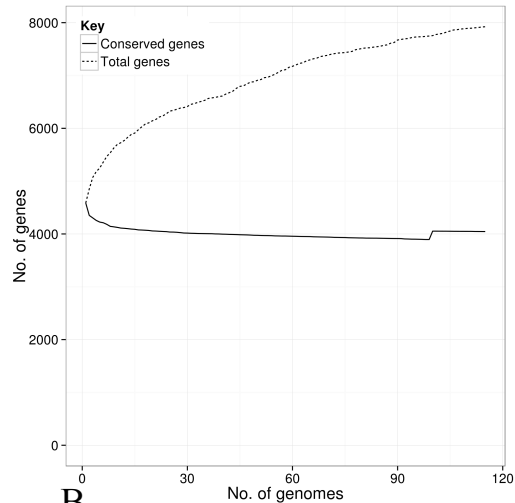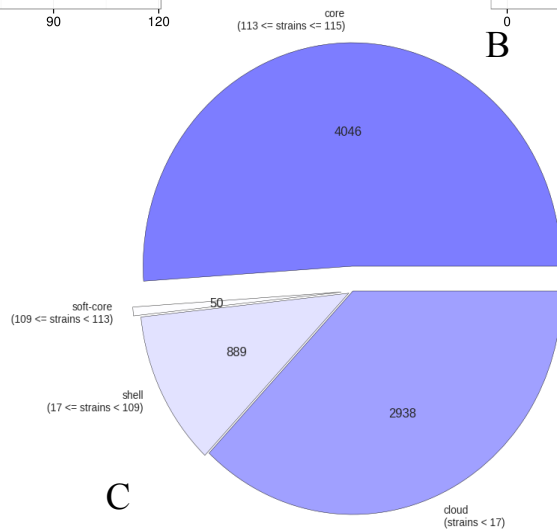

Supplement: S2 Fig — a) Plot showing variance in the number of unique genes found in a single isolate only and the number of new genes as genomes are added to the pan genome. b) Variance in the total number of predicted CDSs (genes) in the pan genome and the of conserved CDSs (99% of isolates) in the core genome as samples are added. c) Breakdown of the frequency of gene in isolates and in the overall collection of S. Weltevreden. Here, the core genome is defined by genes present in 99–100% of isolates, the soft-core by 95–99%, the shell by 15–95% and the cloud by 1–15%. (PDF) [file pntd.0004446.s004.pdf]
